# DreamerNav: learning-based autonomous navigation in dynamic indoor environments using world models

**DOI:** 10.3389/frobt.2025.1655171

**Published:** 2025-09-26

**Authors:** Stuart Shanks, Jonathan Embley-Riches, Jianheng Liu, Andromachi Maria Delfaki, Carlo Ciliberto, Dimitrios Kanoulas

**Affiliations:** Robot Perception and Learning Lab, Intelligent Robotics Research Line, Department of Computer Science, University College London, London, United Kingdom

**Keywords:** autonomous navigation, world model reinforcement learning, dynamic obstacle avoidance, quadrupedal robots, path planning

## Abstract

Robust autonomous navigation in complex, dynamic indoor environments remains a central challenge in robotics, requiring agents to make adaptive decisions in real time under partial observability and uncertain obstacle motion. This paper presents DreamerNav, a robot-agnostic navigation framework that extends DreamerV3, a state-of-the-art world model–based reinforcement learning algorithm, with multimodal spatial perception, hybrid global–local planning, and curriculum-based training. By formulating navigation as a Partially Observable Markov Decision Process (POMDP), the system enables agents to integrate egocentric depth images with a structured local occupancy map encoding dynamic obstacle positions, historical trajectories, points of interest, and a global A* path. A Recurrent State-Space Model (RSSM) learns stochastic and deterministic latent dynamics, supporting long-horizon prediction and collision-free path planning in cluttered, dynamic scenes. Training is carried out in high-fidelity, photorealistic simulation using NVIDIA Isaac Sim, gradually increasing task complexity to improve learning stability, sample efficiency, and generalization. We benchmark against NoMaD, ViNT, and A*, showing superior success rates and adaptability in dynamic environments. Real-world proof-of-concept trials on two quadrupedal robots without retraining further validate the framework’s robustness and quadruped robot platform independence.

## 1 Introduction

Robot navigation in increasingly complex and dynamic environments remains a central challenge in autonomous robotics. We define complexity to encompass the number and density of obstacles, the diversity of their motion patterns, and the overall size and geometry of the operational space. Ensuring efficient, adaptable, and safe navigation is essential for real-world deployment, particularly in indoor environments such as warehouses, homes, and offices. These settings frequently feature dynamic elements, such as moving people, moving equipment, and shifting objects, which pose significant challenges to reliable robot operation. In such scenarios, the navigation system must operate under *partial observability* and *uncertain dynamics*, making the ability to predict and adapt to changes critical to success.

Path planning, a key component of autonomous navigation, is typically categorized into static and dynamic approaches. Static methods such as curve fitting, grid-based search, and optimization-based planning rely on complete environmental knowledge to generate feasible offline paths. These methods are effective in structured, predictable environments but struggle to adapt to unforeseen changes or moving obstacles, limiting their applicability in real-time, dynamic contexts. Dynamic path planning approaches operate online using real-time sensor data, enabling reactive behavior in partially observable environments. Common methods include artificial potential fields (APF), the dynamic window approach (DWA), and reinforcement learning (RL) Fox et al. (1997). Although these methods improve adaptability, they often suffer from limitations such as local minima, convergence instability, and high computational overhead. Furthermore, most existing methods oversimplify robot dynamics by treating the robot as a holonomic point mass, disregarding real-world constraints such as actuator limits, non-holonomic motion models, and the need for predictive reasoning in the presence of moving obstacles.

Optimal control-based methods offer a promising direction by explicitly incorporating motion dynamics and physical constraints, such as MPPI Williams et al. (2018), U-MPPI Mohamed et al. (2025b), MPCC Brito et al. (2019), C2U-MPPI Mohamed et al. (2025a), SH-MPC de Groot et al. (2025), and DRL-VO Xie and Dames (2023). These approaches formulate navigation as an optimal control problem (OCP), enabling solutions that respect robot kinematics and environmental constraints. However, they often suffer from the “curse of dimensionality,” making them computationally impractical for environments with multiple agents or moving obstacles. Classical planners such as A* Hart et al. (1968), widely used in static map-based navigation Duchoň et al. (2014); Tang et al. (2021); Wang et al. (2011), also fall short in dynamic environments due to their reliance on static global maps and limited capacity for online re-planning. More recently, advances in machine learning have introduced model-free reinforcement learning methods such as Deep Q-Networks (DQN) Mnih et al. (2013) and Proximal Policy Optimization (PPO) Schulman et al. (2017), which have shown success in navigation tasks Han et al. (2020); Wenzel et al. (2021); Georgakis et al. (2022). However, these approaches typically require large volumes of training data, exhibit high sample inefficiency, and are sensitive to reward design, making them less suitable for real-time deployment in safety-critical environments.

Hybrid approaches such as PRM-RL Faust et al. (2018) combine sampling-based planning with RL for long-range navigation but rely on pre-computed static roadmaps, limiting adaptability. Visual navigation methods like ViNT Shah et al. (2023b) use subgoal-based planning and large vision models to guide goal-conditioned navigation, but struggle with undirected exploration and computational demands. NoMaD Sridhar et al. (2023) introduces a unified policy for both goal-directed and undirected navigation, but remains limited in its ability to predict the trajectories of moving obstacles, an essential capability for safe, proactive real-time decision-making.

This study is motivated by the need to improve autonomous robot navigation in dynamic indoor environments, particularly by enhancing the robot’s ability to integrate *local egocentric sensing* with *allocentric spatial context*, predict obstacle motion, and adapt its path in real time. Although recent work Liang et al. (2018) has made progress in this direction, challenges remain in achieving generalization across quadruped robots and ensuring robust collision avoidance in diverse environments. To address these limitations, we propose DreamerNav, a robot-agnostic navigation framework that builds on DreamerV3 Hafner et al. (2023), a state-of-the-art world model–based reinforcement learning algorithm, and extends it with.

1. Multi-modal observation integration — combining egocentric depth images with a structured local occupancy map containing dynamic obstacle positions, historical trajectories, points of interest, and a static A* path for global guidance;
2. Hybrid global–local planning — using the A* path to maintain global optimality while DreamerV3’s latent-space policy adapts locally to dynamic obstacles;
3. Curriculum-based training — gradually increasing task difficulty to improve learning stability and generalization;
4. Sparse yet informative reward shaping — balancing safety, efficiency, and goal completion;
5. Quadruped robot independence and sim-to-real transfer — demonstrated through deployment on two distinct quadrupedal robots without retraining.

Unlike its NVIDIA Isaac Gym counterpart NVIDIA (2024), which focuses on parallelized RL at scale, NVIDIA Isaac Sim NVIDIA (2023) offers high-fidelity physics and photorealistic rendering, ideal for perception-rich training. DreamerV3’s Recurrent State-Space Model (RSSM) encodes both stochastic latent states and deterministic history, enabling the system to model environmental uncertainty and perform long-horizon latent rollouts for decision-making under partial observability Hafner et al. (2023); Wu et al. (2023). In our design, the robot is abstracted as a floating-body cognitive planner, separating high-level decision-making from low-level control. This abstraction ensures quadruped robot independence and supports deployment across different robot types.

In summary, this work presents a generalized, sample-efficient, and robust navigation framework that integrates learned environmental dynamics, real-time planning, and high-fidelity simulation to bridge the gap between perception, planning, and control in mobile robotics. We benchmark against state-of-the-art visual navigation methods and a classical planner in both single- and multi-goal dynamic navigation tasks, and validate the approach with preliminary real-world tests. The remainder of the paper is structured as follows: Section 2 reviews relevant literature on world models and navigation; Section 3 details our POMDP formulation, world model architecture, and observation/action spaces; Section 4 presents the curriculum training procedure; Section 5 reports experimental results in simulation and real-world trials; and Section 6 concludes with discussion, limitations, and future directions.

## 2 Related work

### 2.1 World models

There is a longstanding hypothesis that humans and animals develop an internal model of the world—often referred to as a world model—to predict how environments behave Land (2014). These models typically operate over abstract and compressed representations of the world, capturing concepts and their relationships, which supports generalization across new environments governed by similar dynamics Forrester (1971). In reinforcement learning (RL), world models refer to generative models that probabilistically map events to outcomes Friston et al. (2021). For simplicity, we consider model-based RL algorithms to be synonymous with world models, as they map an agent’s state and action to predicted future states and rewards Thrun et al. (1990).

Model-based RL constructs an internal model of environment dynamics to guide decision-making and policy optimization. This contrasts with model-free RL, where learning occurs purely from interaction without explicitly modeling the environment. World models offer a sample-efficient pathway for control policy learning by enabling agents to simulate trajectories within a learned internal model. Notably, recent work has shown that world models can achieve state-of-the-art performance across a variety of control tasks and complex environments Huang et al. (2025); Hansen et al. (2024); Lancaster et al. (2024); Burchi and Timofte (2025); Shah et al. (2024); Jamgochian et al. (2023); Brohan et al. (2023). Furthermore, their generalization abilities have been validated in real-world deployments Wu et al. (2023).

Two leading world model approaches in recent literature are TD-MPC2 Hansen et al. (2024) and DreamerV3 Hafner et al. (2023). TD-MPC2, an extension of TD-MPC Hansen et al. (2022), integrates trajectory optimization with model predictive control (MPC), learning an internal dynamics model and optimizing over a planning horizon to select control actions. It excels at high-frequency control adjustments, making it particularly effective in rapidly changing environments. However, its reliance on repeated optimization can impose significant computational burdens, limiting real-time scalability.

In contrast, DreamerV3 offers a flexible, scalable solution for model-based RL, building upon earlier work (PlaNet Hafner et al. (2019b), DreamerV1 Hafner et al. (2019a), DreamerV2 Hafner et al. (2020)). It employs a Recurrent State-Space Model (RSSM) Hafner et al. (2019b); Karl et al. (2016) to encode sequences of pixel-based or scalar-based observations—via CNNs or MLPs—into a compact latent space. A transition model predicts future states purely in the latent space, and observations are reconstructed using a decoder, similar to a Variational Autoencoder (VAE) Kingma and Welling (2013), providing rich learning signals. An actor-critic architecture is used to optimize policies based on imagined rollouts in latent space. DreamerV3 stands out due to its generality and ability to handle diverse inputs and environments. Its latent-space planning allows for real-time adaptation, such as updating predictions of dynamic obstacles and re-planning paths accordingly. These features make it particularly suited for autonomous navigation in dynamic indoor environments, where flexibility and sample efficiency are critical. While TD-MPC2 is effective for fine-grained control, DreamerV3 offers a more scalable and adaptable framework. We therefore opt to use DreamerV3—specifically, a PyTorch implementation of the most recent version NM512 (2023)—in this work, exploring its suitability for solving navigation tasks via predictive world modeling.

### 2.2 Robot navigation

Autonomous robot navigation has long been a central challenge in robotics, particularly in dynamic, real-world environments. Traditional approaches typically rely on mapping and planning based on geometric data Marder-Eppstein et al. (2010); Macenski et al. (2020). These methods, while robust and interpretable, often ignore semantic or visual information that can be captured through rich sensor modalities such as RGB or depth cameras.

With the advent of deep learning, the problem has increasingly been framed as a data-driven task. AI-based approaches have employed large-scale multimodal foundation models to derive control policies from sensory input Firoozi et al. (2023); Shah et al. (2024), promoting generalization across environments and tasks. Our work is inspired by such AI-driven strategies, yet operates at a smaller scale, focusing on learning-based visual navigation without the need for billion-parameter models. A key aspiration in generalized visual navigation is zero-shot adaptability to novel environments, using only egocentric sensory observations. Toward this goal, world models have emerged as a promising solution. Models like DreamerV3 Hafner et al. (2023); Huang et al. (2025) enable learning predictive representations of environmental dynamics in latent space, facilitating adaptive planning and decision-making in real time. Incorporating collision avoidance directly into the reward function further improves safety in complex settings.

Several recent approaches have attempted to bridge the gap between simulation and real-world deployment, either by training on real-world datasets Shah et al. (2023a); Loquercio et al. (2018), or leveraging sim-to-real transfer Anderson et al. (2020); Kadian et al. (2020); Truong et al. (2024); Stamatopoulou et al. (2025). Among them, ViNT (Visual Navigation Transformer) Shah et al. (2024) stands out for its ability to generalize across robots and environments, fine-tuned with minimal data. Similarly, RT-1 Brohan et al. (2023) demonstrates large-scale policy learning using transformers for real-world robot control.

Recent research has also investigated hybrid and bio-inspired approaches to navigation. Dey et al. Dey et al. (2024) propose a hierarchical framework that dynamically switches between classical planners and learned neural planners, enabling robots to leverage the complementary strengths of both paradigms and improving performance in real-world deployment. In parallel, advances in vision-language navigation extend the scope of robot autonomy beyond geometric reasoning. Du et al. Du et al. (2024) introduce VL-Nav, a real-time system that fuses pixel-wise vision-language features with spatial reasoning, achieving robust navigation to human-specified targets while operating at 30 Hz on resource-constrained hardware. Beyond engineering-driven approaches, neuroscience-inspired models have also contributed to the field. Nakashima et al. Nakashima et al. (2024) present a hippocampal formation-inspired framework for global self-localization, integrating recurrent state-space models with Monte Carlo localization to recover from the kidnapped robot problem. Their results suggest that sparse, place-cell-like latent representations can enhance resilience in self-localization. Together, these lines of work demonstrate the value of combining classical planning, multimodal grounding, and biologically inspired models in advancing autonomous navigation.

#### 2.2.1 Navigation in dynamic environments

Dynamic environments—such as warehouses, hospitals, or urban spaces—introduce an additional layer of complexity, requiring real-time perception, localization, path planning, and decision-making Zhu and Zhang (2021). Depth cameras are particularly valuable for capturing spatial information for obstacle detection and navigation Ellis et al. (2022). In simulation, pre-built 2D maps and depth sensors enable decision-making. Transitioning to the real world, SLAM systems incorporating LiDAR, IMUs, and ceiling-mounted vision sensors become essential for dynamic obstacle tracking and map building.

#### 2.2.2 Localization and mapping

Traditional SLAM techniques (e.g., EKF-SLAM Dissanayake et al. (2001), FastSLAM Montemerlo et al. (2002)) struggle in dynamic settings. Enhanced methods like DynaSLAM Bescos et al. (2018), Bescos et al. (2021) use deep learning to differentiate between static and dynamic elements, while FAST-LIO2 Xu et al. (2022) integrates LiDAR and IMU for robust positioning in real time.

#### 2.2.3 Local path planning

Local path planning must make instantaneous decisions to avoid dynamic obstacles. Three broad categories of methods are typically used: 1) Reactive Methods like Dynamic Window Approach (DWA) Fox et al. (1997) and Vector Field Histogram (VFH) Borenstein et al. (1991). 2) Predictive Methods use models like Social Force Models (SFMs) Helbing and Molnar (1995) or Gaussian Processes (GPs) Mehta et al. (2018). 3) Learning-Based Methods, such as PRM-RL Faust et al. (2018), ViNT Shah et al. (2024), and NoMaD Sridhar et al. (2023), integrate planning with deep learning. Recent work has extended local planners with terrain-aware perception for legged and wheeled robots Liu et al. (2024); Stamatopoulou et al. (2024), improving safety in unstructured and dynamic environments. Our approach falls within this category, leveraging DreamerV3 to anticipate future states and adapt plans in latent space.

#### 2.2.4 Global path planning

For long-range tasks, global planning ensures an optimal path from start to goal: 1) Classical Methods such as A* Hart et al. (1968) and Dijkstra’s algorithm DIJKSTRA (1959). 2) Sampling-based Approaches like PRM Kavraki et al. (1996), RRT LaValle (1998), and RRT* Karaman and Frazzoli (2011). 3) Learning-based Methods Hamandi et al. (2019); Qureshi et al. (2019); Ichter and Pavone (2019).

In our framework, the global path is computed using A* and provided as an observation. DreamerV3 then uses this to inform real-time local decisions, combining the global optimality of A* with dynamic adaptability to short-term environmental changes.

### 2.3 Simulators for training RL agents in robotics

Simulators are essential for training RL agents, especially in robotics, where real-world training could be slow, expensive, and risky. Simulators offer a controlled environment in which agents can learn or test strategies, accelerating iteration and development.

Gazebo Koenig and Howard (2004), PyBullet Ellenberger (2018) and MuJoCo (Multi-Joint dynamics with Contact) Todorov et al. (2012) are some of the most popular simulators in the robotic society. Gazebo is the most flexible and has some of the most realistic physics. It supports a wide range of sensors and models of robots. It also offers great integration with ROS. However, for complex environments, this may get computationally intensive and slow down the simulations. PyBullet is popular because it is much easier to use and more integrative with Python, making it popular in RL research. In contrast, it has lower physical fidelity compared to Gazebo and MuJoCo. MuJoCo is the best regarding detailed and accurate physical interactions in such applications as robotic locomotion and manipulation and allows high-speed simulations along with accurate modeling. Nonetheless, these simulators may fail to meet real requirements concerning computation necessities and complexity for high-fidelity tasks.

Isaac Sim Mittal et al. (2023) is developed by NVIDIA and is outstanding in its use of GPU computing for high-fidelity simulation. It provides realistic physics, photo-realistic rendering, and integration with NVIDIA’s AI framework. Isaac Sim is highly scalable and can be integrated efficiently with machine learning frameworks. Although it requires significant computational resources, its advanced features and powerful capabilities for processing complex dynamic environments make it ideal for our work. Its high-fidelity simulations accurately model real-world environments, and its efficient integration with ML frameworks supports the development and testing of complex RL algorithms. These features make Isaac Sim particularly suitable for training autonomous navigation systems that must adapt to dynamic changes, ensuring that our methods are fully ready for practical deployment.

## 3 Methodology

We propose a world-model-based local navigation framework for autonomous robots in dynamic indoor environments, using DreamerV3 as a predictive planner. Our system enables goal-directed navigation while adapting to the presence of moving obstacles, such as humans or equipment in warehouses and offices. The key innovation lies in combining DreamerV3’s latent-space dynamics modeling with multimodal inputs to support reactive but goal-aligned behavior.

### 3.1 Problem formulation

The navigation task is modeled as a partially observable Markov decision process (POMDP) defined by the tuple (S,H,A,P,R,O,γ) , where S is the stochastic state space, representing the latent state of the environment, which includes essential dynamic information about obstacles and the robot’s position; H is the deterministic history space, where past observations and actions are stored, providing contextual information that aids in overcoming partial observability; A denotes the action space, which includes discrete robot actions such as moving forward, turning, or stopping; P represents the state transition model, governing the probabilistic transitions between states based on actions; R is the reward function, providing feedback that guides the robot’s behavior towards the navigation goal while avoiding collisions; O defines the observation space, consisting of sequences of image and vector data, representing both visual and spatial information about the environment; γ is the discount factor, balancing immediate rewards with future gains.

At each time step t , the robot receives a sequence of K observations $\left(o_{t},o_{t+1},\ldots ,o_{t+K}\right)$ , where each $o_{t+\tau }$ with {τ∈Z∣0≤τ≤K} contains both image and vector data, capturing the current observation information. These high-dimensional observations are decoded into a stochastic latent state $s_{t+\tau }$ , which encapsulates essential features of the environment with a reduced set of variables. The latent state $s_{t+\tau }$ provides a compressed yet informative representation of the current situation in the environment. The agent accumulates historical information in the form of a deterministic feature history $h_{t+\tau }$ , which aggregates past observations and actions to provide context for decision making under partial observability. The deterministic feature history $h_{t+\tau }$ ensures that the model retains memory of past events, which is critical for effective planning. Given the current action $a_{t+\tau }$ , the state transition model P predicts the next stochastic latent state $s_{t+\tau +1}$ and updates the deterministic history feature $h_{t+\tau +1}$ . The policy π , which maps states to actions, aims to maximize the expected cumulative reward by optimizing the value function $v_{\pi }\left(s_{t}\right)$ , defined in Equation 1 as:

$$v_{\pi }\left(s_{t}\right)=E_{\pi }\left[\overset{\infty }{\underset{i=0}{\sum }}\gamma^{i}r_{t+i}|s_{t}\right]$$ (1)

where $r_{t+i}$ denotes the immediate reward at time step t+i . The objective is to learn an optimal policy π* that maximizes this value function, allowing the robot to navigate dynamically, avoid obstacles, and efficiently reach its destination. A full explanation of all terms in the POMDP formulation is found in Table 1.

**TABLE 1:** Notation used in the POMDP formulation of the navigation task.

Symbol	Description
S	Stochastic state space (latent states of environment: obstacles, robot position)
H	Deterministic history space (past observations and actions for context)
A	Action space (discrete robot actions: move forward, turn, stop)
P	State transition model (probabilistic transitions given actions)
R	Reward function (feedback for reaching goal and avoiding collisions)
O	Observation space (sequences of image and vector data)
γ	Discount factor (trade-off between immediate and future rewards)
$o_{t}$	Observation at time t (image and vector data)
$s_{t}$	Stochastic latent state at time t (compressed environment features)
$h_{t}$	Deterministic feature history at time t (aggregated memory of past)
$a_{t}$	Action taken at time t
$r_{t}$	Immediate reward at time t
$v_{\pi }\left(s_{t}\right)$	Value function under policy π
π	Policy mapping states to actions
π*	Optimal policy maximizing expected return

### 3.2 World model

Our navigation framework is based on a world model, based on the DreamerV3 architecture, which learns to predict environment dynamics in a latent space. This model enables the agent to simulate future trajectories and plan actions without direct interaction with the environment. Using these internal simulations, the agent can make informed and sample-efficient decisions in complex dynamic settings. The world model is a deep neural network that captures the underlying dynamics of the environment by learning from the agent’s experiences stored in a replay buffer. The model is trained to predict future states, rewards, and other critical elements necessary for effective navigation. Table 2 summarizes the notation used in the world model formulation.

**TABLE 2:** Notation used in the DreamerNav world model (RSSM) formulation.

Symbol	Description
$x_{t}$	Raw observation at time t (depth image, occupancy map, $q_{t}$ vector inputs)
${\hat{x}}_{t}$	Reconstructed observation from decoder network
$q_{t}\in R^{k}$	Vector observations at time t (e.g., goal (x,y) , heading, orientation, distance-to-next goal)
$a_{t}$	Action taken by the agent at time t
$r_{t}$	Immediate reward at time t
${\hat{r}}_{t}$	Predicted reward from reward network
$z_{t}$	Stochastic latent state at time t (compressed representation of $x_{t}$ )
$h_{t}$	Deterministic hidden state at time t (recurrent memory)
${\text{enc}}_{\theta }$	Encoder mapping $\left(x_{t},h_{t-1},a_{t-1}\right)\mapsto z_{t}$
${\text{dyn}}_{\theta }$	Dynamics predicting $z_{t+1}$ given $\left(z_{t},h_{t},a_{t}\right)$
f	Recurrent update function for $h_{t}$
${\text{dec}}_{\theta }$	Decoder reconstructing ${\hat{x}}_{t}$ from $z_{t}$
${\text{rew}}_{\theta }$	Reward network predicting ${\hat{r}}_{t}$ from $z_{t+1}$
θ	Learnable parameters of the world model
*Replay buffer*	Storage of past trajectories (x,a,r) for training
*Loss*	Sum of reconstruction and prediction errors (states/rewards)

**FIGURE 1:**
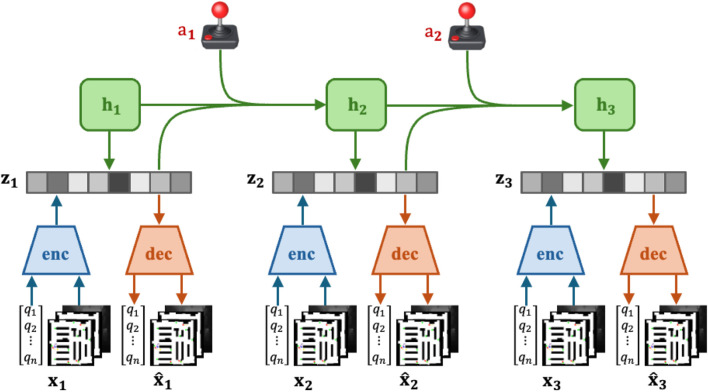
Scenario 3: Navigation in a confined space with multiple dynamic obstacles. All methods fail to reach the goal. Our method makes limited avoidance adjustments and collides with a moving obstacle. NoMaD and ViNT take large detours but still collide, reflecting overly conservative avoidance. A* fails due to its static path intersecting a moving obstacle.

Figure 1 illustrates the Recurrent State-Space Model (RSSM) structure used in our approach. Each observation $x_{t}$ consists of multimodal inputs: the vector observation $q_{t}$ (goal coordinates, heading, orientation, distance to next goal), the global occupancy map overlaid with dynamic obstacles and points of interest, and the egocentric depth image from the robot’s camera. Observations are then encoded into a latent state $z_{t}$ using the encoder network. The dynamics network then predicts the next latent state $z_{t+1}$ based on the current latent state, action, and hidden state. Finally, the decoder reconstructs the original observation ${\hat{x}}_{t}$ , allowing us to visualize and verify the predictions of the model. To handle high-dimensional sensory inputs, such as occupancy maps and depth images, the world model leverages a Recurrent State-Space Model (RSSM). The RSSM consists of the following components, each represented by a distinct neural network.

#### 3.2.1 Encoder network

The encoder network, denoted as ${\text{enc}}_{\theta }$ , encodes the multimodal observation $x_{t}$ (e.g., occupancy maps and depth images) into a lower-dimensional stochastic latent state $z_{t}$ as shown in Equation 2:

$$z_{t}∼{\text{enc}}_{\theta }\left(x_{t},h_{t-1},a_{t-1}\right)$$ (2)

where $h_{t-1}$ is the hidden deterministic state of the previous time step and $a_{t-1}$ is the action taken by the agent at the previous time step. Each observation consists of low-dimensional vector inputs (e.g., goal coordinates, heading, orientation, distance to the next goal), the global occupancy map overlaid with dynamic obstacles and points of interest, and the egocentric depth image from the robot’s camera. This process reduces the complexity of the inputs while retaining the essential features needed for state prediction.

#### 3.2.2 Dynamics network

The dynamics network, denoted ${\text{dyn}}_{\theta }$ , predicts the next latent state $z_{t+1}$ based on the current latent state $z_{t}$ shown in Equation 3, the hidden deterministic state $h_{t}$ and the action $a_{t}$ at shown in Equation 4:

$$z_{t+1}∼{\text{dyn}}_{\theta }\left(z_{t},h_{t},a_{t}\right)$$ (3)

$$h_{t}=f\left(h_{t-1},z_{t},a_{t}\right)$$ (4)

where f is a recurrent function that updates the hidden deterministic state $h_{t}$ based on the previous hidden state $h_{t-1}$ , the latent state $z_{t}$ and the action $a_{t}$ . This network models the transition dynamics of the environment.

#### 3.2.3 Decoder network

The decoder network, denoted ${\text{dec}}_{\theta }$ , reconstructs the original sensory inputs $x_{t}$ from the current latent state $z_{t}$ as shown in Equation 5:

$${\hat{x}}_{t}={\text{dec}}_{\theta }\left(z_{t}\right)$$ (5)

Although not directly used for decision making, this network provides a way to inspect and verify the model’s understanding of the environment.

#### 3.2.4 Reward network

The reward network, denoted ${\text{rew}}_{\theta }$ , predicts the immediate reward $r_{t}$ associated with the predicted latent state $z_{t+1}$ as shown in Equation 6:

$${\hat{r}}_{t}={\text{rew}}_{\theta }\left(z_{t+1}\right).$$ (6)

This network is crucial for the agent’s policy optimization, as it estimates the expected outcome of each action.

The world model is optimized using stochastic backpropagation, with the objective of minimizing the reconstruction loss and prediction errors for future states and rewards. By training these components jointly, the model becomes a powerful simulator that predicts the outcomes of actions efficiently, enabling the agent to plan and act in dynamic environments without direct interaction.

### 3.3 Actor-critic learning

**FIGURE 2:**
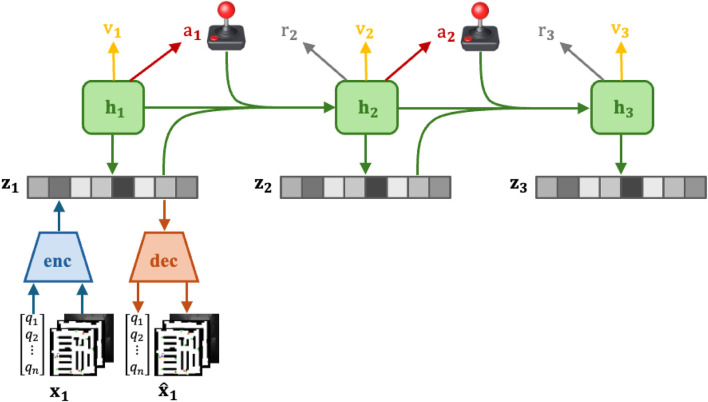
Example trajectory of Boston Dynamics Spot executing DreamerNav.

While the world model represents task-agnostic knowledge about the dynamics of the environment, the actor-critic algorithm learns behaviors specific to the task at hand. The actor-critic framework operates in the latent space predicted by the world model, enabling the agent to perform rollouts of future states and evaluate potential actions without directly interacting with the environment. Figure 2 provides an overview of the actor-critic framework in the DreamerV3 model. The world model predicts latent states $z_{t}$ , and the actor network selects an action $a_{t}$ . The predicted rewards $r_{t}$ and reconstructed observations ${\hat{x}}_{t}$ are also shown, which guide the learning process for both the actor and the critic networks.

The actor-critic algorithm consists of two core networks: an actor network $\pi \left(a_{t}|z_{t}\right)$ and a critic network $v\left(z_{t}\right)$ . The role of the actor network is to learn a distribution of actions $a_{t}$ for each latent state $z_{t}$ that maximizes the sum of future predicted rewards. The critic network is trained to predict the sum of future rewards through temporal difference (TD) learning Sutton and Barto (2018). This allows the algorithm to consider rewards beyond a short-term planning horizon and thus learn long-term strategies.

#### 3.3.1 Actor network

The actor network $\pi_{\theta }\left(a_{t}|z_{t}\right)$ selects actions that maximize the expected cumulative rewards. Given a latent state $z_{t}$ , the policy $\pi_{\theta }\left(a_{t}|z_{t}\right)$ produces a distribution over actions $a_{t}$ , from which the action is sampled:

$$a_{t}∼\pi_{\theta }\left(a_{t}|z_{t}\right)$$ (7)

The actor in Equation 7 is trained to maximize the λ returns, which are calculated to handle the trade-off between bias and variance. According to Hafner et al. (2023), Hafner et al. (2020), Hafner et al. (2019a), the λ -returns $V_{t}^{\lambda }$ are defined as shown in Equation 8:

$$V_{t}^{\lambda }≐r_{t}+\gamma \left(\left(1-\lambda \right)v\left(s_{t+1}\right)+\lambda V_{t+1}^{\lambda }\right),$$ (8)

where $r_{t}$ is the reward at time t , γ is the discount factor and $v\left(s_{t+1}\right)$ is the estimate of the value of the next state. The actor’s goal is to select actions that maximize these λ -returns in future trajectories.

#### 3.3.2 Critic Network

The critic network $v_{\theta }\left(z_{t}\right)$ estimates the value of the current latent state $z_{t}$ , which corresponds to the expected sum of future rewards starting from $z_{t}$ as shown in Equation 9:

$$v_{\theta }\left(z_{t}\right)=E_{\pi }\left[\overset{\infty }{\underset{i=0}{\sum }}\gamma^{i}r_{t+i}|z_{t}\right]$$ (9)

The critic is trained using TD-learning to minimize the discrepancy between the predicted value and the computed λ -returns. This is accomplished by minimizing the following loss function as shown in Equation 10:

$$L\left(v_{\theta }\right)=E\left[{\left(v_{\theta }\left(z_{t}\right)-V_{t}^{\lambda }\right)}^{2}\right]$$ (10)

This minimizes the difference between the predicted value and the target value derived from the λ -returns.

The actor and critic are updated using the Adam optimizer. According to Hafner et al. (2023), Hafner et al. (2020), Hafner et al. (2019a), to encourage exploration and prevent the policy from collapsing into a deterministic strategy, an entropy term is added to the actor’s loss function as shown in Equation 11:

$$L\left(\pi \right)≐-E\left[\overset{H}{\underset{t=1}{\sum }}\ln\pi \left(a_{t}|s_{t}\right)\text{sg}\left(V_{t}^{\lambda }-v\left(s_{t}\right)\right)+\eta H\left[\pi \left(a_{t}|s_{t}\right)\right]\right],$$ (11)

where η is the regularization coefficient of the entropy, encouraging explorfation by maintaining high entropy in the policy.

Additionally, following Hafner et al. (2023), Hafner et al. (2020), Hafner et al. (2019a) in computing the λ -returns, we use a slowly updated copy of the critic network, a common technique that improves the stability of learning. Importantly, the gradients from the actor and critic networks do not influence the world model, preventing overly optimistic updates to the model predictions.

### 3.4 World model hyperparameters

In this section, we present the key hyperparameters used in our DreamerV3-based navigation framework. These hyperparameters were selected to optimize both the world model and the actor-critic components for efficient learning and robust performance in dynamic environments. Table 3 summarizes the primary hyperparameters in the General, World Model, Actor-Critic, and Optimizer categories.

**TABLE 3:** Key hyperparameters for the world model, actor-critic, and optimizers.

Name	Symbol	Value
General		
Total Steps	—	$5\times 10^{5}$
Replay Capacity (FIFO)	—	$10^{6}$
Batch Size	B	16
Batch Length	T	32
Replay Ratio	—	0.5
Start Learning Steps	—	$10^{4}$
Activation Function	—	LayerNorm + SiLU
World Model		
RSSM Size	—	768
MLP Size	—	4×768
Recurrent State Size	—	2048
Discrete Size	—	32
Stochastic Size	—	32
KL Dynamic	—	0.5
KL Regularizer	—	1.0
Actor Critic		
Imagination Horizon	H	15
Discount Factor	γ	0.996997
Return Lambda	λ	0.95
Entropy Coefficient	—	0.0003
All Optimizers		
World Model Learning Rate	—	$10^{-4}$
World Model Adam Epsilon	ε	$10^{-6}$
Actor Learning Rate	—	$8.0\times 10^{-5}$
Actor Adam Epsilon	ε	$10^{-5}$
Gradient Clipping	—	1,000

The hyperparameters listed in Table 3 were chosen based on empirical testing and optimization to balance model accuracy, stability, and computational efficiency. For the world model, we utilize a Recurrent State-Space Model (RSSM) with a size of 768 and a multilayer perceptron (MLP) size of 4×768 units, ensuring sufficient capacity for encoding environment dynamics. The recurrent state size of 2048 provides additional memory for handling partial observability. The stochastic and discrete latent sizes are set to 32, balancing complexity and computational cost. The terms of regularization of the KL divergence, specifically the dynamic KL coefficient of 0.5 and the regularization coefficient of 1.0, help prevent overfitting and encourage generalization. These values were selected to ensure that the world model learns robust latent representations while effectively capturing the dynamic aspects of the environment.

For the actor-critic framework, an imagination horizon of 15 steps was used, allowing the agent to simulate future trajectories and make long-term decisions. The discount factor γ is set to 0.996997, promoting future rewards while focusing on immediate goals. The entropy coefficient of 0.0003 encourages exploration, preventing premature convergence to suboptimal policies. The optimization settings include a learning rate of $10^{-4}$ for the world model and $8.0\times 10^{-5}$ for the actor, with Adam epsilon values of $10^{-6}$ and $10^{-5}$ , respectively. These values were tuned for stable convergence. Gradient clipping at 1,000 was used to prevent exploding gradients during training, ensuring stable and efficient learning.

Overall, these hyperparameters are fine-tuned to balance the trade-offs between performance, exploration, and computational efficiency, resulting in a reliable navigation system that can handle dynamic environments with continuous learning and adaptation.

### 3.5 Observations

The observations used by the agent are divided into two main categories: image observations and vector observations. These observations provide the agent with essential spatial information about the environment, enabling it to navigate through dynamic and static obstacles in the warehouse effectively. The following sections detail the components of each observation type and their role in the decision-making process.

#### 3.5.1 Image observations

**FIGURE 3:**
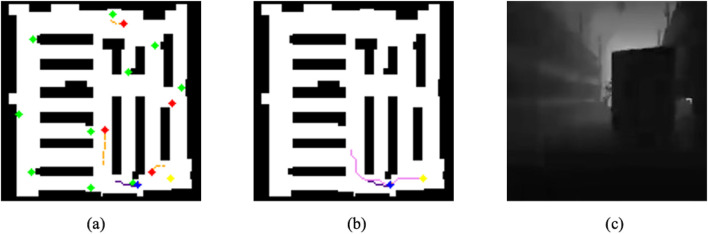
DreamerV3 RSSM Architecture: each observation $x_{i}$ includes vector observations q (such as x-y goal coordinates, heading robot direction, robot orientation, and distance to the next goal), as well as occupancy global maps of the environment overlaid with dynamic obstacles in red and points of interest in green, as well as the current egocentric depth image from the robot’s camera.

The image observations include two main components: occupancy map observations and egocentric depth images. The occupancy map observations consist of two layers, each providing crucial insights into the current state of the environment. These observations are initially provided as single-channel images, but can be visualized in multiple color channels for clarity, as depicted in Figure 3.

Table 4 provides a detailed description of the different elements of the map represented in the map observations. Each row describes a specific type of object or space in the environment, along with the corresponding color used to visualize it in the observation map.

**TABLE 4:** Map observation: Elements and color representation.

Map element	Color	Description
Empty Space	White	Free space where the agent can navigate freely
Static Obstacles	Black	Static Objects that cannot be traversed
Dynamic Obstacles	Red	Moving objects that the agent must avoid
Dynamic Obstacle Trajectory	Orange	The recent path of dynamic obstacles
Positions of Interest	Green	Locations frequently visited by dynamic obstacles
Agent	Blue	The current position of the agent within the map
Agent Trajectory	Indigo	The recent path the agent has traveled
Agent Orientation	Coral	The direction the agent is currently facing
A* Path	Violet	The optimal A* path to the goal
Goal	Yellow	The target goal that the agent is trying to reach

The first map layer visualizes the agent’s position, orientation, and past trajectory while simultaneously displaying static and dynamic obstacles. This provides the agent with crucial contextual awareness of its surroundings. The second map layer overlays the precomputed A* path, which is calculated at the start of each episode and remains static throughout. This path helps the agent navigate toward its goal by following the optimal route through the warehouse. The A* path is based on the static map of the warehouse and the obstacle configuration at the beginning of the episode, offering an efficient navigation strategy without continuous updates. The final goal location is represented in yellow.

The depth image adds another layer of information, helping the agent perceive the distance to nearby objects in the environment. This input is particularly valuable for obstacle avoidance, as it allows the agent to sense dynamic changes in real time, adjusting its actions accordingly to avoid collisions.

The combination of map layers and depth images provides the agent with a rich representation of both static and dynamic elements within the environment. The color-coded map layers allow for better visual interpretation and debugging, while the depth images enable real-time obstacle avoidance. Together, these image observations play a critical role in equipping the agent to navigate complex environments, ensuring it can make informed decisions based on its surroundings.

#### 3.5.2 Vector observations

In addition to image-based inputs, the agent also relies on vector observations that capture specific, detailed spatial relationships. These vectors provide additional information that complements the map and depth images, offering more precise details on the agent’s position, heading, orientation, and distance to its goal.

Table 5 outlines the key vector observations that provide the agent with structured information about its position, orientation, and relationship to its sub-goal. Each observation is described in terms of its shape and function in the navigation process, offering insights into how the agent makes decisions based on both its own state and its goal.

**TABLE 5:** Vector observations of the agent.

Observation	Shape	Description
Goal	(4)	Represents the relative position to the next subgoal and the direction to that subgoal (relative x, y, and orientation)
Heading	(2)	Describes the direction to the goal relative to the agent’s current facing orientation (cosine and sine of the angle)
Orientation	(2)	Captures the agent’s current orientation using the cosine and sine of the orientation angle
Distance	(1)	The distance from the agent to the next subgoal in meters

The goal vector contains the relative x and y coordinates to the next subgoal on the A* path, along with information about the orientation (cosine and sine of the direction). The subgoal is dynamically updated based on the agent’s position and proximity to the A* path. As the agent approaches a subgoal, it updates to the next subgoal along the A* path. This process ensures that the agent follows a smooth and optimal route to its final destination, adjusting when obstacles are encountered. The heading vector provides the agent with its directional heading toward the goal relative to its current orientation. By calculating the cosine and sine of the angle between the forward direction of the agent and the goal, the agent can adjust its rotation to stay on track. This heading vector is crucial for maintaining the correct orientation as the agent follows the A* path. The orientation vector represents the current facing direction of the agent. It is expressed using the cosine and sine of the orientation angle, helping the agent align its movement with its intended path. This vector helps to turn and adjust the agent’s rotation to ensure smooth navigation. The distance vector is a scalar value that provides the agent with the distance to the next sub-goal in meters. Knowing the distance allows the agent to adjust its speed or planning strategy accordingly. Shorter distances may prompt the agent to slow down for precise obstacle avoidance, while longer distances enable faster, more direct movement.

Together, these vector observations form a comprehensive set of inputs that complement the image observations. They provide the agent with precise and detailed information necessary for following the A* path and avoiding static and dynamic obstacles. This multimodal observation space enhances the agent’s ability to navigate complex environments, making informed decisions in real time based on both visual and spatial inputs.

### 3.6 Actions

In our framework, the agent operates in a discrete action space where each action corresponds to a specific movement or rotation. The agent selects four possible actions at each time step that determine how it moves within the environment. Table 6 summarizes the discrete actions available that control the agent’s navigation behavior.

**TABLE 6:** Agent’s discrete action space with linear and angular velocities.

Action	Linear velocity	Angular velocity
Move Forward	1 m/s	0 rad/s
Turn Left	0 m/s	$\frac{\pi }{2}\text{ rad/s}$
Turn Right	0 m/s	$-\frac{\pi }{2}\text{ rad/s}$
No Movement	0 m/s	0 rad/s

The discrete action space ensures that the agent has simple, yet effective control over its movements, enabling it to navigate the environment efficiently. Each action is designed to correspond to basic movements that are appropriate for the warehouse environment, balancing simplicity and effectiveness.

The agent operates at a control frequency of 10 Hz, which means that it makes decisions and executes actions 10 times per second. This high frequency enables the agent to react quickly to dynamic obstacles and changes in the environment, ensuring smooth and responsive navigation. The linear velocity of the agent is set at 1 m per second, allowing it to move forward at a consistent speed that is suitable for the warehouse environment: fast enough for efficient navigation but slow enough for precise control. In addition, the agent rotates at an angular velocity of $\frac{\pi }{2}$ radians per second, allowing it to make quick and sharp turns without overshooting its target orientation. Finally, the agent-mounted camera is positioned at a height of 1 m, providing a realistic perspective for perceiving obstacles and features in the environment, such as shelves, dynamic obstacles, and pathways. These parameters work together to ensure that the agent can navigate complex environments both efficiently and safely.

### 3.7 Rewards

The rewards of our navigation framework are designed to balance efficiency in achieving goals with safe obstacle avoidance. Through extensive testing and reward engineering, we have developed a reward scheme that encourages the agent to prioritize efficient navigation while avoiding collisions. The overall reward for each step is calculated as the sum of individual rewards as defined in Equation 13:

$$r_{t}=r_{\text{goal}}+r_{\text{collision}}+r_{\text{heading}}+r_{\text{movement}}+r_{\text{time}}+r_{\text{subgoal}}+r_{\text{overlay}}$$ (13)

Where each component serves a specific purpose in guiding the agent’s behavior. Table 7 summarizes the rewards, their formulas, and their effects on agent behavior.

**TABLE 7:** Summary of rewards and their formulas.

Reward	Formula	Description
Goal Reward	$r_{\text{goal}}=100$	Given when the agent reaches the goal
Collision Penalty	$r_{\text{collision}}=-50$	Applied when the agent collides
Heading Alignment	$r_{\text{heading}}=\cos\left(\theta_{\text{goal}}\right)$	Encourages aligning its heading with goal
Movement Penalty	$r_{\text{movement}}=-1$	Penalizes the agent for minimal movement
Time Penalty	$r_{\text{time}}=-0.5$	Encourage faster goal-reaching
Subgoal Progress	$r_{\text{subgoal}}=5$	Reward for reaching a subgoal on the A* path
Overlay Penalty	$r_{\text{overlay}}=-1.0$	Proximity to obstacles on the maps

The goal reward of 100 is awarded when the agent successfully reaches its destination, providing a strong motivation for goal-reaching behavior. This reward incentivizes the agent to navigate effectively through the environment to achieve its objective. Collision penalties of −50 discourage risky movements and collisions with obstacles. This penalty helps to train the agent to be cautious around both static and dynamic obstacles, encouraging safe navigation practices. The heading alignment reward, calculated as the cosine of the angle between the current direction of the agent and the goal, encourages the agent to maintain the right orientation. This reduces unnecessary turns and ensures that the agent follows a more direct path toward its target. To prevent stagnation, a movement penalty of −1 is applied if the agent does not move sufficiently over a period of time. This penalty ensures that the agent maintains a steady pace, avoiding idle behavior during navigation. The time penalty of −0.5 per step motivates the agent to reach the goal quickly, promoting time-efficient navigation. In dynamic environments, this encourages the agent to make quick decisions and adapt to changes promptly. Subgoal progress rewards of five are granted when the agent reaches predefined points along the A* path. These rewards guide the agent through optimal intermediate waypoints, keeping it on track toward the final goal. Finally, the overlay penalty of −1.0 penalizes the agent for getting too close to obstacles, encouraging it to maintain a safe distance. This helps prevent collisions and ensures smoother navigation through the environment.

This reward structure is the culmination of extensive testing and iterative reward engineering. Through numerous experiments, we have carefully balanced various reward components to encourage the agent not only to reach its goal efficiently but also to prioritize safe and smooth navigation. By penalizing collisions and rewarding successful subgoal achievements, the system guides the agent to avoid obstacles while minimizing unnecessary movements or deviations. The combined rewards for heading accuracy, goal proximity, and smooth movement ensure that the agent navigates complex and dynamic environments with an optimal balance between speed, safety, and precision.

## 4 Curriculum training

### 4.1 Training setup

Curriculum training is a crucial aspect of our navigation framework, aimed at progressively increasing the difficulty of the navigation task. The motivation behind this approach is to allow the agent to explore and learn from a balanced proportion of successful and unsuccessful trajectories. By starting with simpler tasks and gradually increasing difficulty, the agent can develop a more robust navigation policy, maintaining consistent learning throughout the training process.

Through extensive experimentation, we have fine-tuned the curriculum levels to ensure that the agent can reliably and stably improve its performance. This approach allows the agent to gradually adapt to more complex scenarios, which is essential to achieve effective navigation in dynamic environments. In the training process, we included four dynamic obstacles to simulate the movement of workers in a factory warehouse. To improve training efficiency, we employ eight agents in the environment, each isolated from others to prevent collisions. For each episode, a random goal is generated for each agent at the beginning. When an agent collides or reaches the maximum step number, the episode ends. If an agent reaches the goal, the episode continues with a new randomly generated goal until a collision or maximum step number is reached. Throughout this process, we monitor each agent for collisions with static and dynamic obstacles. Upon collision, the episode ends and the agent is reset to a random position.

The curriculum training consists of six levels, each designed to incrementally increase task difficulty. The parameters for each level of the curriculum are described in Table 8. In each level, the agent is required to navigate to goals within a specified distance range from its current position. The step number defines the maximum steps allowed per episode and the distance to the goal determines how close the agent must get to the goal to consider it reached. Advancing to the next curriculum level requires the agent to complete 20 episodes with a success rate of at least 0.75 in the last 20 episodes. This ensures that the agent has sufficiently mastered the current difficulty level before moving on to a more challenging one.

**TABLE 8:** Parameters for each curriculum level.

Curriculum	Goal distance range	Max steps	Goal reaching radius
1	[2.0,5.0]	200	1.4
2	[5.0,8.0]	250	1.3
3	[8.0,12.0]	300	1.2
4	[12.0,16.0]	350	1.1
5	[16.0,20.0]	400	1.0
6	[2.0,∞]	400	1.0

### 4.2 Training process

Training was carried out using the designed curriculum on a system equipped with an NVIDIA 4090 GPU with 24 GB of memory. The training process lasted 24.79 h, covering a total of 495,000 policy steps. The training parameters are summarized in Table 3.

**FIGURE 4:**
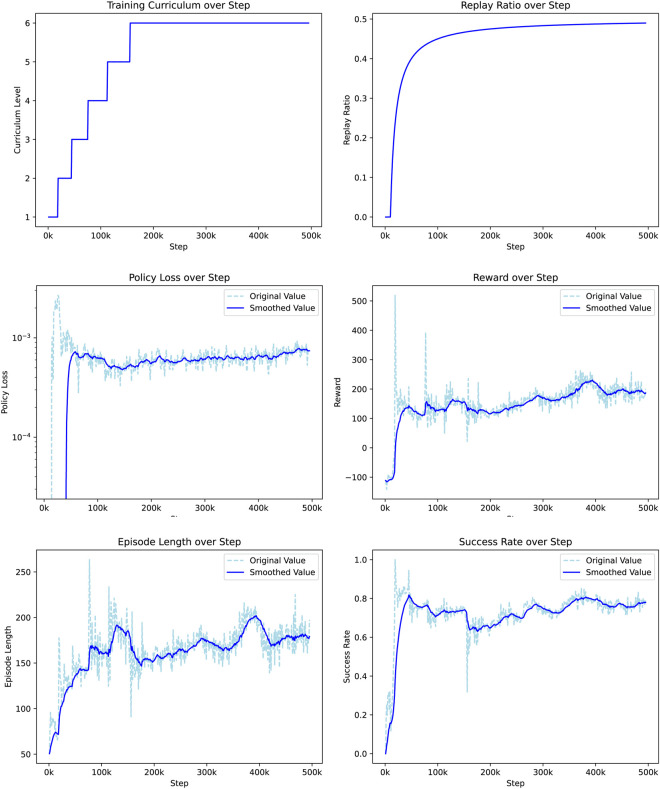
DreamerV3 actor-critic framework.

Figure 4 illustrates the training metrics. The first plot shows the progression of the curriculum level, indicating that the agent successfully mastered each level, ultimately reaching the sixth level of the curriculum. This progression confirms that the agent consistently met the performance criteria for the advancement of complexity. The replay ratio curve represents the proportion of training data sampled from the replay buffer. Initially set at zero, it gradually increased to stabilize at 0.5, reflecting an effective balance between new experiences and previously collected data during training. The plot of policy loss, which initially decreases rapidly, indicates a quick improvement in policy learning. After 100k steps, the learning rate slows down, but the overall trend remains indicative of ongoing policy refinement. The reward curve starts at −100, indicating that random exploration yields a low reward. After 50k steps, the reward stabilizes above 100 and approaches 200 at the end of training. This implies that on average, the agent can reach approximately two goals per episode, demonstrating significant progress in navigation efficiency. The episode-length plot starts at around 50 steps, with an upward trend within each curriculum level. This suggests that the agent’s ability to avoid obstacles and extend its navigation duration improves with time. In particular, the length of the episode decreases with each increase in the difficulty of the curriculum, confirming that the task becomes more challenging as the agent progresses. Finally, the success rate curve shows a rapid increase early in the training, surpassing 0.8 within the first 50k steps. Each increase in curriculum difficulty results in an initial drop in success rate, followed by recovery and improvement. The agent achieves a final success rate of 78% at the highest difficulty level, indicating robust navigation performance even in challenging randomized goal scenarios. The correlation of success rate with policy loss suggests a consistent learning process throughout the curriculum levels.

## 5 Experiments

This section presents the experimental evaluation of the proposed navigation framework in a simulated factory warehouse environment developed in NVIDIA Isaac Sim. The objective is to evaluate the effectiveness of the framework in dynamic and cluttered indoor settings through single-goal and multi-goal navigation tasks. We compare our method against three established baselines—NoMaD, ViNT, and A*—to demonstrate its performance in terms of adaptability, efficiency, and goal-directed behavior under realistic but controlled conditions.

We note that this evaluation is intended as a proof-of-concept rather than a large-scale validation. Although our approach has been successfully transferred between two quadrupedal robot platforms in controlled settings demonstrating qudruped robot independence within this scope, we do not claim broad generalization across robot types, terrains, or large-scale deployments. Similarly, scalability to larger fleets or outdoor environments is discussed as a promising direction for future work, rather than as a demonstrated capability in the present study.

**FIGURE 5:**
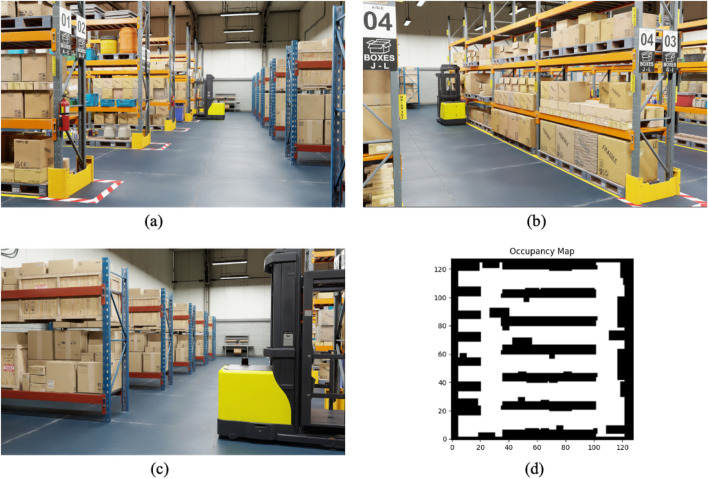
Map layers and depth image. **(a)**Map layer 1. **(b)**Map layer 2. **(c)**Depth image.

The factory warehouse environment was constructed within Isaac Sim, a high-fidelity simulation platform that provides realistic physics and environmental modeling. The simulation scene measures 32×32 meters and contains typical warehouse elements, including shelves, forklifts, tables, and boxes. These objects serve as static obstacles around which the robot must navigate to reach its destination. The warehouse layout, as shown in Figure 5, presents a challenging geometry for the navigation task, with narrow aisles and cluttered areas requiring precise obstacle avoidance. The warehouse occupancy map represents the spatial configuration and distribution of obstacles in a 2D grid format, which the agent uses to inform its path planning and obstacle avoidance strategies.

**FIGURE 6:**
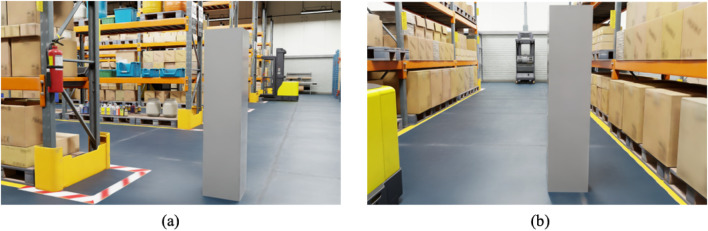
Training metrics over policy step, illustrating the progression of curriculum levels, policy loss, reward, episode length, and success rate.

**FIGURE 7:**
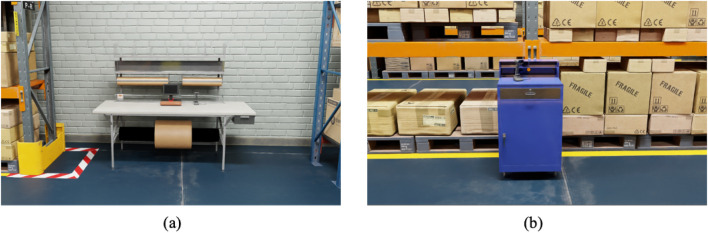
Training warehouse environment with occupancy map. **(a)** Training environment (view 1). **(b)** Training environment (view 2). **(c)** Training environment (view 3). **(d)** Occupancy map of training environment.

In addition to static obstacles, dynamic objects are introduced to simulate moving elements within the environment, mimicking workers in a real factory setting. These dynamic obstacles are represented by simple cuboid shapes, as illustrated in Figure 6, due to time constraints and the need for efficient simulation. These cuboid blocks move through the scene, traveling between predefined *Points of Interest* (POIs), which represent key locations where workers might operate, such as tables and cabinets as shown in Figure 7. The dynamic obstacles follow A*-based navigation to move between POIs, ensuring realistic and unpredictable interactions with the robot.

The agent’s task is to navigate from a starting point to a goal location while avoiding static and dynamic obstacles. The simulation environment was designed to provide diverse and realistic challenges, combining static and dynamic elements to mimic real-world conditions in warehouses or industrial settings. The training process was conducted using only a single simulated warehouse environment. Although this setup provided meaningful results, future work could benefit from the introduction of multiple different environments or the application of domain randomization techniques to improve the generalization of the navigation system. By diversifying training environments, the agent’s robustness and ability to generalize in different settings and obstacle configurations could be further enhanced. This will allow the system to perform more reliably in new or unseen real-world scenarios.

In order to test the efficiency of the navigation framework, the robot is mounted with an Intel RealSense D435i depth camera inside Isaac Sim. The motion of the robot and its perception are managed via a fully implemented ROS stack in Isaac Sim. This stack comprises several nodes that drive communication and control: Subscribe to velocity commands, compute odometry, and publish odometry data. The Isaac Compute Odometry Node estimates the position and orientation of the robot, while the ROS Publish Odometry and the ROS Publish Raw Transform Tree node broadcast localization and mapping data. In all, these allow the robot to efficiently receive, process, and execute navigation instructions. This setup with ROS ensures that different navigation methods may be tested and integrated under the same conditions. The agent’s control settings including the control frequency, maximum linear velocity, and maximum angular velocity are identical to those used during training.

A novel factory warehouse environment in Isaac Sim constitutes the evaluation environment, different from that used in training. It is also 32 × 32 m but arranged differently with respect to the shelves and obstacles, as shown in Figure 9. This layout has been specially designed with challenging scenarios for navigation algorithms, including narrow passages and unevenly distributed obstacles. Floor colors are varied throughout the environment so that vision-based methods, such as ViNT and NoMaD, can operate reliably. The colored floor helps maintain stability in their visual input, enabling effective navigation across different parts of the environment.

**FIGURE 8:**
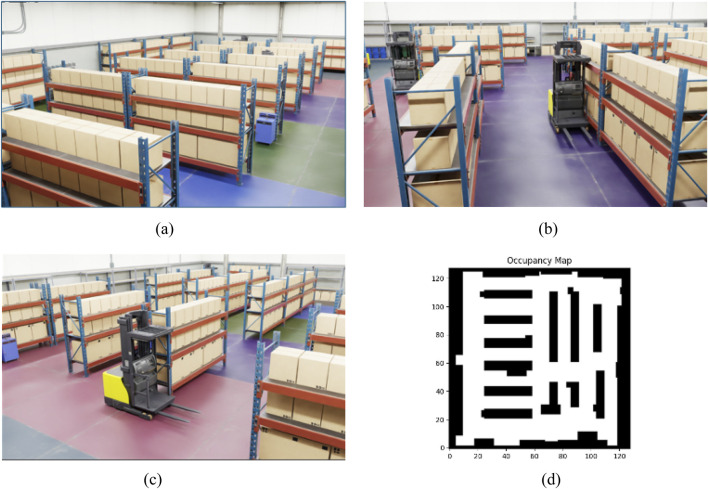
Dynamic obstacles in the simulation environment. **(a)** Dynamic object 1. **(b)** Dynamic object 2.

### 5.1 Baseline navigation methods

Under the same conditions, we tested four methods: our proposed method, NoMaD Sridhar et al. (2023), ViNT Shah et al. (2023b), and A* Hart et al. (1968). Each method was deployed in the environment using the ROS stack to control the robot. NoMaD combines goal-directed navigation with exploration in one framework. In our experiments, we provide NoMaD with the RGB images of the target location to provide input for guided robot navigation. This enables the robot to navigate towards the target based on visual input while avoiding obstacles. ViNT is designed to generalize across different environments using a Transformer-based architecture. For this method, we provided RGB images sampled every 1 m along the precomputed A* path from the start to the goal. These images are used to construct a topological map that helps the robot navigate following this path while avoiding obstacles. The A* method directly computes the shortest path on a grid-based map, considering static obstacles and the initial positions of dynamic obstacles. The robot is then controlled to move along this pre-planned A* path to the goal. Unlike our method and the others tested, A* does not adapt to changes in the environment in real-time, making it more suitable as a baseline for comparison.

### 5.2 Experiment 1: single-goal navigation

For a fair comparison of the agent’s ability to navigate to only one goal location in the presence of dynamic obstacles, 25 different experiments with random seeds were conducted. Each seed defines a unique starting position, goal position, and dynamic obstacle path, providing a robust test of the agent’s adaptability and efficiency in dynamic environments. This section compares every method against their capability to steer towards a pre-defined location while avoiding obstacles. In all trials, the starting A* distance from agent to goal was 18.56 m on average. The results of this experiment are summarized in Table 9. Column titles have been abbreviated in Table 9. Succ Rate is the number of successful goal achievements over the total trials. Avg Dist (m) and Avg Time (s) is the total distance and time over all trials. Succ Dist (m) and Succ Time (s) are the average distance and time over all successful trials.

**TABLE 9:** Performance comparison in single-goal navigation.

Method	Succ	Avg	Avg	Succ	Succ
	Rate	Dist (m)	Time (s)	Dist (m)	Time (s)
Our Method	0.72	20.38	25.89	23.67	28.02
NoMaD	0.64	26.24	38.59	31.09	45.90
ViNT	0.56	20.98	31.13	24.15	33.32
A*	0.48	15.92	21.55	19.77	26.47

Our method reached the highest success rate of 0.72, showing great navigation with the avoidance of obstacles in dynamic scenes. The average travel distance of the agent for all trials is 20.38 m, reaching 23.67 m for only those trials where navigation was successful. It indicates that while navigating successfully, the agent often detours extra to avoid dynamic obstacles, which usually makes it longer but safer to the goal. The average in all trials was 25.89 s, but increased to 28.02 s for successful trials, reflecting the additional time required to perform avoidance maneuvers.

NoMaD has an overall success rate of 0.64, proving that it manages dynamic environments, but slightly lower than our method. The higher mean travel distance for all trials was 26.24 m, while that of successful trials was 31.09 m, reflecting its more exploratory approach due to the diffusion policy, which aims at flexibility and undirected exploration. The longer navigation times are again due to the previous one, with 38.59 s being the average for all trials and 45.90 s in the successful ones. The strong increase, yet in distance as well as in time, for successful trials testifies to the fact that rather often the exploration carried out by NoMaD results in less efficient paths, whereas it eventually reaches the goal. ViNT yields a success rate of 0.56, which, considering its vision-based navigation strategy, is reasonable. On average, it travels 20.98 m in all trials, increasing to 24.15 m in successful trials. This would indicate that ViNT adjusts its path to avoid obstacles, but is burdened by dynamic updates owing to its reliance on static visual cues. The average time taken in all trials is 31.13 s, increasing to 33.32 s in the successful trials. The longer times compared to our method may indicate that, while avoiding obstacles, ViNT takes more cautious or indirect routes, reducing its efficiency. The A* algorithm, which only succeeded in 0.48 of all runs, best illustrates the difficulty of performing static path planning in dynamic environments. Meanwhile, its average travel distance is the shortest among all trials and successful trials, 15.92 and 19.77 m, respectively. That reflects A*‘s strict adherence to the precomputed shortest path but inability to adapt to moving obstacles, leading to frequent failures.

The average time across all trials was 21.55 s, but this rate increases to 26.47 s in the trials where A * reached the goal. The increased time due to temporary obstructions suggests that even when A* reaches the goal, it cannot proactively adapt its path. Together, the added data show that while our method may involve longer travel distances and times in successful trials compared to A*, it achieves a much higher overall success rate. The increased distance and time hint at the agent’s adaptability and proactive obstacle avoidance, which are critical for safe navigation in dynamic environments. Our method effectively balances path efficiency with the flexibility required to handle dynamic obstacles for improved performance.

This experiment underscores the importance of adaptation in dynamic environments: Although A* offers the shortest path in static situations, without runtime adjustments, it results in a lower success rate. NoMaD and ViNT demonstrate trade-offs between exploration-oriented vs efficiency-oriented approaches: NoMaD prioritizes exploration at the expense of longer paths and times; ViNT relies heavily on visual cues, but lacks dynamic path adjustment. Our method’s ability to adapt its path in real time, while maintaining relative efficiency, highlights its effectiveness in navigating dynamic obstacles.

### 5.3 Experiment 2: multi-goal navigation

We designed an omnigoal experiment to test the capability of the agent in complex navigation, where an agent is supposed to reach five different goals placed randomly in sequence inside the environment. This simulates scenarios such as a robot performing a series of tasks on either a warehouse or factory floor in a dynamically changing environment. Each episode runs until the agent has reached all five goals or collided with some obstacle. This experiment measures the agent’s ability to manage sequential navigation tasks, where both the path of the agent and the positions of obstacles change continuously.

We ran five trials, each with a different random seed defining initial positions, goal locations, and dynamic obstacle paths. The total distance of A * from the five goals was averaged at 96.43 m. The results are detailed in Table 10.

**TABLE 10:** Performance comparison in multi-goal navigation.

Method	Avg goals	Avg dist (m)	Avg time (s)
Our Method	3.2	68.21	82.55
NoMaD	2.6	78.91	98.36
ViNT	1.8	46.30	58.24
A*	1.2	31.78	44.17

In Table 10, Avg Goals is the average number of goals successfully reached per episode. Avg Dist (m) denotes the average total travel distance in meters per episode, and Avg Time (s) is the average total time in seconds taken per episode. Our approach achieves much higher performance for the multi-goal navigation task, resulting in an average of 3.2 goals per episode, which is the highest among all methods.

This would mean that the agent has learned to navigate through new goal positions while efficiently avoiding dynamic obstacles. The average travel distance of 68.21 m, though shorter than the total A* path distance of 96.43 m, reflects the fact that often, the agent does not reach all five goals due to episode termination upon collision. On the other hand, the higher number of goals reached signifies better adaptability and robustness in dynamic environments. NoMaD reaches goals with reasonable competence in complex navigation tasks, averaging 2.64. Its longer average travel distance and time at 78.91 m and 98.36 s, respectively, show that its style of navigation is more explorative in nature. That means that although NoMaD has capable dynamic environment navigation, its diffusion-based policy results in less efficient paths and increased exposure to dynamic obstacles. So, it will reach fewer goals compared to our method.

The average of 1.8 goals per episode reached by ViNT indicates that the agent is ill-equipped for dynamic or sequential tasks. Having no large world model and relying purely on visual inputs, it is not well-positioned to adapt to real-time adjustments in general. The shorter average travel distance of 46.30 m and a time of 58.24 s should indicate that ViNT often terminates episodes early due to collisions and therefore fails to reach more goals. This limit underscores the increased need for adaptability in dynamic environments. The A* approach reaches a maximum average of only 1.2 goals per episode, showing the limits of static path planning in dynamic multi-goal environments. Its deterministic nature, along with an inability to adapt to moving obstacles, generates numerous collisions and early terminations of episodes. The shortest distance traveled on average, 31.78 m, could be completed in 44.17 s and is indicative not of efficiency but of the agent’s inability to advance further due to collisions.

This experiment underscores the importance of adaptability and real-time decision making in multi-goal navigation tasks in dynamic environments. The integration of our method with a world model and curriculum training enhances its ability to handle dynamic changes, leading to superior performance compared to those of the other methods. The limitations identified in NoMaD, ViNT, and A* emphasize the challenges between overexploring methods, methods lacking adaptability, and methods incapable of adapting to dynamic obstacles. These results highlight the need to integrate dynamic planning capabilities into navigation algorithms to manage evolving environments effectively.

### 5.4 Navigational behaviors in representative scenarios

To better understand their performance in each situation, we have chosen three representative scenarios from Experiments 1 and 2. These will help us analyze more precisely the strengths and weaknesses of each method in different navigation tasks. In each scenario, we have taken three key scenarios for each method showing their strategies and behaviors. The meaning of pixels is explained in Table 4.

#### 5.4.1 Scenario 1: clear path navigation

**FIGURE 9:**
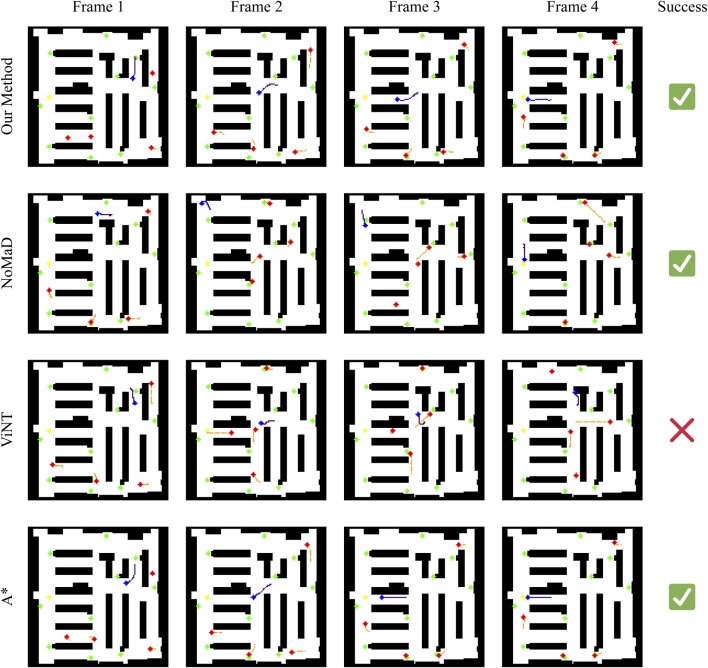
Points of interest (POIs) in the environment. **(a)** Position of interest (table). **(b)** Position of interest (cabinet).

In the first scenario in Figure 9, the robot is tasked with navigating a relatively clear path, with minimal interference from dynamic obstacles. Our method, NoMaD, and A* successfully reach the goal, while ViNT fails due to a collision with a static obstacle. Our method closely follows the A* path, maintaining an efficient and direct route towards the goal. This indicates that in situations where the environment is relatively static or predictable, our method can achieve near-optimal navigation, comparable to A*‘s precomputed path. However, it offers the advantage of real-time adaptability should unexpected changes occur.

**FIGURE 10:**
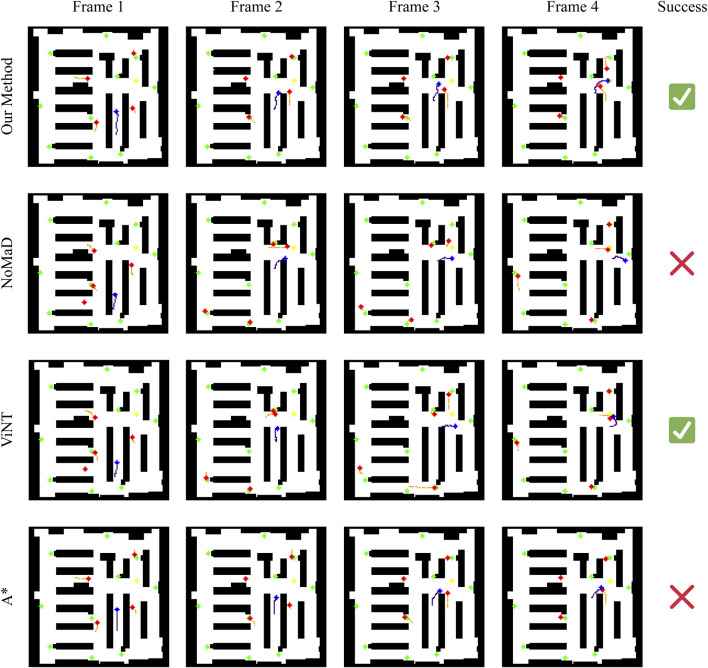
Overview of the evaluation warehouse environment in isaac sim. **(a)** Evaluation environment (view 1). **(b)** Evaluation environment (view 2). **(c)** Evaluation environment (view 3). **(d)** Occupancy map of evaluation environment.

Whereas NoMaD ultimately succeeds, it does so by a more circuitous route compared to our method, A*, which shows NoMaD’s intrinsic exploration strategy. In this case, the intrinsic exploration strategy leads to taking less direct routes even when more straightforward paths are available. Although this characteristic is helpful in complex and uncertain settings, it results in unnecessary travel and reduced efficiency in simpler scenarios like this one. ViNT exhibits a significant deviation in behavior compared to the other methods. Initially it moves slowly, thus a dynamic obstacle comes into its path. Instead of navigating around this obstacle within the original path corridor, ViNT opts for a large detour. This overly cautious avoidance strategy ultimately leads to a collision with a static obstacle, demonstrating a potential limitation in ViNT’s decision-making process. This outcome suggests that ViNT may struggle in environments where a quick and efficient response to obstacles is required, favoring large detours over minor adjustments that could preserve the initial path.

#### 5.4.2 Scenario 2: Obstacle-rich path navigation

**FIGURE 11:**
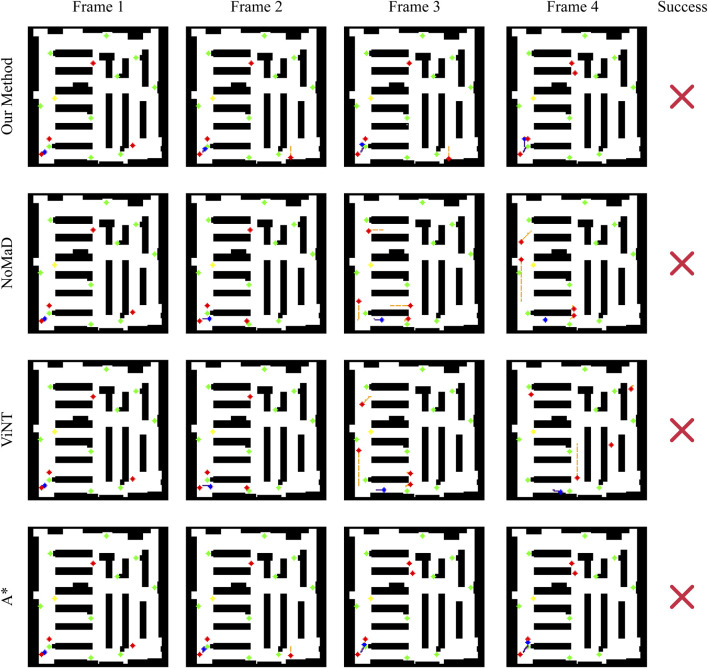
Scenario 1: Navigation in a relatively clear environment. Our method and A* reach the goal directly, with our method matching A*‘s efficiency while remaining adaptable in real time. NoMaD succeeds but takes a longer route due to its intrinsic exploration strategy. ViNT takes a large detour, ultimately colliding with a static obstacle.

A further complicated case arises in the second scenario in Figure 11, when active obstacles appear in the robot’s path. In this situation, our approach reacts efficiently by performing a left maneuver to avoid the moving obstacle approaching from the right. The agent maintains an efficient and adaptive trajectory, deviating only slightly from the planned path to avoid the obstacle. This behavior highlights the strength of our method in dynamically adapting to the environment while minimizing deviations from the optimal route.

NoMaD shows a conservative avoidance strategy. It makes a huge turn away from the goal direction when encountering the dynamic obstacle and deviates into an exploration phase. This overcautious approach causes the robot to stray far from the target and fail to recover to the path. This highlights a limitation in NoMaD’s policy: imprecise adjustments around obstacles, leading to inefficient navigation. ViNT performs a relatively successful avoidance maneuver in this scenario. It turns right to avoid a dynamic obstacle on the left and then reorients itself towards the goal. However, this approach involves a more complex sequence of turns compared to our method, resulting in a longer travel path. ViNT’s strategy indicates an ability to navigate around obstacles effectively, but with a tendency to overcomplicate its trajectory, which can result in longer travel times and less efficient navigation. A* fails in this scenario due to its reliance on a precomputed path that does not adapt to dynamic changes. The appearance of a dynamic obstacle on its path results in a collision, underscoring a fundamental limitation of static path planning algorithms in dynamic environments. This failure emphasizes the need for real-time adaptability in navigation systems, particularly in environments with unpredictable elements.

#### 5.4.3 Scenario 3: Complex and confined space

The third scenario shown in Figure 11 represents a highly challenging scenario where the robot is initialized in a confined corner with a number of dynamic obstacles around it. This is meant to test each method’s competency in navigating through a tightly bound space with few maneuver options. For this particular case, all four methods fail to reach the goal, but their behaviors can provide insight into their underlying decisions.

In contrast, our approach tries to avoid the obstacles by avoiding them to the left. However, the correction is not enough; thus, the robot collides with a moving obstacle. This reflects that, although our method can make real-time adjustments, it may not be good enough in situations that require an aggressive avoidance action, especially when one is in a confined space with limited escape routes.

NoMaD again demonstrates its overly conservative approach: it finds dynamic obstacles in its view, makes a huge detour, turning to another corridor from the goal, probably leading the robot to stop further, either because of the lack of clear path exploration or simply due to an overly conservative avoidance policy. It seems that NoMaD could use a strategy that would more balance contradictory demands, thus providing a more decisive motion for the robot in complex situations. ViNT shows a similar trend to NoMaD: it takes a detour and eventually collides with a static obstacle. This result highlights a potential weakness in ViNT’s obstacle avoidance strategy, where a wide turn to avoid an obstacle may lead to an incorrect path choice, especially in environments with limited maneuvering options. A* cannot solve this scenario, since it is of a static nature. The route planned in advance goes right through the moving obstacle; a collision is thus impossible to avoid. This, therefore, points to the fundamental limits imposed by static path planning within highly dynamic and confined environments, where adaptability and real-time decision making will be at the heart of navigating such scenarios with success.

### 5.5 Challenges and methodological insights

Together, these scenarios give a very fine comparison of the strengths and weaknesses of each method in various navigation contexts. Our approach strikes a balance between efficient path-following and dynamic obstacle avoidance. It adapts to changes in the environment, maintaining a trajectory close to the optimum path, especially in real-time decision-making scenarios, as there are elements at play. However, in highly complex, confined spaces with more aggressive strategies, it does have some limitations.

NoMaD tends to be more exploratory and conservative in navigation. Although this approach works well in environments where the optimal path is unclear, it can cause inefficiency or failure in straightforward or highly dynamic scenarios. Its tendency to take large detours and enter exploration phases leads to longer travel distances and lower success rates, particularly when quick and precise adjustments are needed. ViNT demonstrates a conservative avoidance strategy; sometimes this leads to excessive detours or even wrong path choices because of the degree of caution applied. Although such a conservative strategy can be much more effective in avoiding any immediate collisions, it is likely to result in inefficient navigation and may fail in scenarios where a more direct reaction to obstacles becomes crucial. Performance via the ViNT in tight spaces hints at the necessity for razor-sharp decision making that balances prudence with the need for efficient path-following. A* serves as a baseline static path planning method, excelling in environments where the path is clear and unchanging. However, its inability to adapt to dynamic obstacles is a significant drawback in environments where real-time changes are frequent. The failures observed in these scenarios highlight the limitations of relying solely on precomputed paths in dynamic settings.

In general, the insights gained from these scenarios suggest that our method offers a promising balance of efficiency and adaptability in dynamic environments. However, there is room for further improvement, particularly in handling complex and confined spaces. Incorporating elements from other methods, such as NoMaD’s exploratory capabilities or ViNT’s cautious avoidance, could potentially enhance our system’s robustness and versatility in diverse navigation tasks.

### 5.6 Preliminary real-world experiments

**FIGURE 12:**
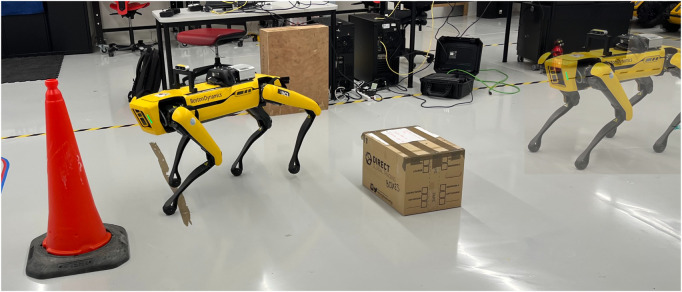
Scenario 2: Navigation with dynamic obstacles. Our method adapts in real time, avoiding a moving obstacle with minimal deviation from the optimal route. NoMaD overreacts, strays far from the goal, and fails to recover. ViNT avoids the obstacle successfully but takes a longer, more complex path. A* collides due to its inability to adapt to dynamic changes.

**FIGURE 13:**
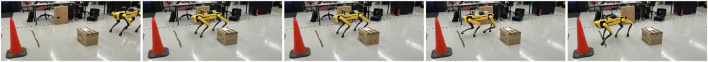
An example of the agent (SPOT) navigating around an obstacle and heading towards the goal.

**FIGURE 14:**
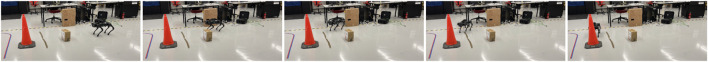
An example of the agent (A1) navigating around an obstacle and heading towards the goal.

To transfer to the real world, we copy the model over to a Robot Operating System (ROS) stack that utilizes velocity-based movement. Sensors similar to those of the virtual ones are used as input into the model running in an ROS node. The output of the model is then taken and converted to these velocity commands and sent to the robot. Two basic scenarios utilizing two different qudruped robotic platforms were conducted using cardboard boxes as obstacles and a traffic cone as the target, as well as using YOLO for object detection of the goal object. Both platforms use the same policy. The task was for the robots to navigate close to the goal without colliding with any of the obstacles. We show this in Figures 12, Figures 13, 14.

## 6 Conclusion

In this work, we have developed and implemented a novel navigation framework for autonomous robots operating in dynamic indoor environments, using DreamerV3 Hafner et al. (2023) as the backbone. By meticulously constructing the entire pipeline–from the simulation setup in Isaac Sim to the real-time integration of the world model with the agent’s control system–we have demonstrated a comprehensive approach that effectively addresses the challenges of navigation amidst dynamic obstacles.

Our method effectively combines world-model-based reinforcement learning with enhanced observations, enabling the agent to make informed, real-time decisions. The integration of both image and vector observations provides the agent with a richer perception of the environment, facilitating efficient path planning and dynamic obstacle avoidance. The experimental results validate the effectiveness of our approach. Our agent achieved a success rate 72% in navigating to the goal, outperforming state-of-the-art methods such as NoMaD Sridhar et al. (2023) and ViNT Shah et al. (2023b), as well as the baseline algorithm A* Hart et al. (1968). This superior performance highlights our method’s ability to adapt to environmental changes more effectively, balancing efficient path-following with robust obstacle avoidance.

Our contributions to the field are threefold.

1. Development of a World Model-Based Navigation Framework: We introduced a novel framework that leverages DreamerV3 for navigation in dynamic environments, demonstrating its applicability beyond traditional reinforcement learning tasks.
2. Enhanced Observation Integration: By incorporating both image and vector observations, we provided the agent with a comprehensive understanding of its surroundings, improving its decision-making capabilities in complex environments.
3. Empirical Validation in Dynamic Scenarios: Through extensive experiments in simulated warehouse environments with dynamic obstacles, we validated the effectiveness of our approach, showing significant improvements over existing methods.

However, our research also revealed certain limitations. The agent’s obstacle avoidance strategy, while generally effective, sometimes falls short when dealing with rapidly approaching dynamic obstacles. This suggests an opportunity to enhance the agent’s responsiveness and adaptability, particularly in scenarios requiring immediate and aggressive avoidance maneuvers.

To address these limitations and further enhance the framework’s performance and versatility, we propose the following future work directions.

1. Improving Obstacle Avoidance Strategies: We plan to integrate advanced perception and prediction mechanisms, such as incorporating recurrent neural networks (RNNs) or attention mechanisms, to better predict the trajectories of dynamic obstacles. In addition, implementing motion prediction models for obstacles can enable the agent to anticipate movements and plan accordingly.
2. Incorporating Semantic Segmentation: By utilizing semantic segmentation techniques, the agent can distinguish between different types of obstacles (e.g., static objects, moving machinery, humans), allowing for more nuanced navigation strategies. This can improve safety and efficiency by enabling the agent to prioritize the avoidance of certain obstacles over others.
3. Training with Diverse Environments and Domain Randomization: We aim to improve the agent’s generalization capabilities by training it in multiple simulated environments with varying layouts and obstacle dynamics. Employing domain randomization techniques will expose the agent to a wide range of scenarios during training, increasing its robustness in unfamiliar settings.
4. Real-World Implementation and Testing: Finally, we plan to implement our framework on physical robots and test it in real-world indoor environments, such as warehouses or office spaces. This will allow us to assess the practical applicability of our method and make the necessary adjustments based on real-world performance.
5. We also acknowledge that ablation studies would provide valuable insights into the contribution of individual components of our method. We leave such studies to a planned extension of this work.

In conclusion, this work presents a significant advancement in autonomous robot navigation within dynamic environments. By effectively combining world-model-based reinforcement learning with enhanced observational inputs, we have demonstrated a method that not only outperforms existing approaches, but also contributes valuable insights to the field. The outlined future work aims to build upon these findings, address current limitations, and expand the applicability of our framework. Through continued refinement and real-world testing, we aim to develop more advanced and versatile autonomous navigation systems capable of operating safely and efficiently in a variety of dynamic and complex environments.

## Data Availability

The raw data supporting the conclusions of this article will be made available by the authors, without undue reservation.
